# Essential Oil Composition and Physiology of Three *Mentha* Genotypes Under Shaded Field Conditions

**DOI:** 10.3390/plants13223155

**Published:** 2024-11-09

**Authors:** Charlotte Hubert-Schöler, Saskia Tsiaparas, Katharina Luhmer, Marcel Dieter Moll, Maike Passon, Matthias Wüst, Andreas Schieber, Ralf Pude

**Affiliations:** 1 Institute of Crop Science and Resource Conservation—Renewable Resources, Agricultural Faculty, University of Bonn, Klein-Altendorf 2, D-53359 Rheinbach, Germany; kluhmer@uni-bonn.de (K.L.); m.moll@uni-bonn.de (M.D.M.); r.pude@uni-bonn.de (R.P.); 2 Institute of Nutritional and Food Sciences, Food Chemistry, Agricultural Faculty, University of Bonn, Friedrich-Hirzebruch-Allee 7, D-53115 Bonn, Germany; tsiaparas@uni-bonn.de (S.T.); passon@uni-bonn.de (M.P.); matthias.wuest@uni-bonn.de (M.W.); 3 Field Lab Campus Klein-Altendorf, Agricultural Faculty, University of Bonn, Klein-Altendorf 2, D-53359 Rheinbach, Germany; 4 Institute of Nutritional and Food Sciences, Molecular Food Technology, Agricultural Faculty, University of Bonn, Friedrich-Hirzebruch-Allee 7, D-53115 Bonn, Germany; schieber@uni-bonn.de

**Keywords:** photosynthetic active radiation, medicinal and aromatic plants, mint, *Mentha* × *piperita*, *Mentha rotundifolia*, menthol, volatile organic compounds, phenotyping, vegetation indices, photosynthesis

## Abstract

*Mentha* spp. are commonly used for the production of tea and for the extraction of essential oils (EOs). The key factor of mint quality is the content and composition of the EO. Health-promoting compounds such as menthol are desirable, whereas the presence of potentially health-damaging compounds such as menthofuran should be avoided. This study examines the effect of shading on the EO content and composition of three *Mentha* genotypes (*Mentha* × *piperita* ‘Multimentha’, *Mentha* × *piperita* ‘Fränkische Blaue’ and *Mentha rotundifolia* ‘Apfelminze’). The *Mentha* genotypes were cultivated in field trials for two years (2022–2023). Each genotype was shaded with a shading net (50% photosynthetic active radiation (PAR) reduction), and a control without shading was prepared. EO content was determined by steam distillation and EO composition was characterized by GC-MS analysis. Furthermore, biomass, vegetation indices (VIs) and the electron transport rate (ETR) were analyzed. While shading led to higher plant heights, higher EO content and a slightly reduced amount of undesired EO compounds, the unshaded control yielded a higher biomass accumulation. Significant genotypic differences were determined. In conclusion, the benefits of shading depend on the intended use and genotype selection.

## 1. Introduction

The genus *Mentha*, one of the most prominent medicinal plants, is used for tea production and EO extraction [1]. *Mentha* spp. estimated to consist of 20 to 30 species and numerous subspecies, hybrids and other cultivars [2]. It belongs to the Lamiaceae family, is perennial and grows worldwide [3]. Mint leaves are primarily used for both tea production and EO extraction [1]. In 2022, peppermint tea alone accounted for around 13% of herbal and fruit tea sales in Germany [4]. Due to their aromatic and health-promoting properties, EOs are used as flavoring agents and food ingredients, as well as in cosmetic and pharmaceutical industries [5]. The latter exploits the antimicrobial, antiviral and antioxidant effects of EOs extracted from *Mentha* cultivars [1]. EO production is also economically viable. The German Federal Statistical Office forecasts an increase in EO turnover in Germany from EUR 2064.3 million in 2019 up to EUR 2349.4 million by 2025, corresponding to an increase of 12.1% [6].

However, not only the EO content is relevant, but also the EO composition. Peppermint EO consists mainly of monoterpenes [7], specifically menthol (30–55%), moderate proportions of its precursor menthone (14–32%) and low levels of the minor compound pulegone [8]. Because pulegone is metabolized to menthofuran in the liver, whose phase 1 metabolist was shown to have hepatotoxic effects [9], the pulegone content should be as low as possible (≤1% is considered as safe [10]). As the content and composition of EO are quality-determining, optimization is needed. There are genetic, environmental, and management influences, typically abbreviated to G × E × M, that can be used to influence optimization [11]. Genotype selection has a severe influence not only on the EO content but also on the EO composition, depending on which monoterpene is predominant. Therefore, *Mentha* genotypes can be categorized by three metabolic pathways. The menthol pathway with menthol, menthone and menthofuran (e.g., *Mentha* × *piperita* L., *Mentha longifolia* L. or *Mentha arvensis* L.) as the main components of the EO, the carvone pathway with carvone, dihydrocarvone and carveol (e.g., *Mentha spicata* L. or *Mentha* × *rotundifolia* L.) and the linalool pathway with linalool and linalyl acetate (e.g., *Mentha aquatica* var. *citrata* or *Mentha* × *piperita* (L.) Huds.) [3]. Environmental influences on the EO composition can be nutrient availability [12] or irradiation [13]. Management influences include harvest time [14] or post-harvest processes [15]. Management is the most adaptable factor, and is particularly important for responding to increasingly variable weather conditions [16].

Shading regimes [17] can decrease abiotic stress caused by high light intensities belonging to the two categories of management and environmental influence [18]. For example, shading influences evaporation, weed pressure and temperature, and protects the plant from heavy rain or wind [19]. Natural shading may originate from trees (e.g., agroforestry) [20], tall crops or bushes (e.g., intercropping) [19], whereas artificial shading may be accomplished, for example, by agrophotovoltaic [21] or shading nets [22]. Phenotyping can be used to measure such influences. Using hyperspectral vegetation indices (VIs) is a way of non-invasive phenotyping to gather information about plant vitality. These VIs can be divided into three categories: structure, biochemistry and plant physiology/stress [23]. In the physiology category, for example, the VI Photochemical Reflectance Index (PRI) can be found, which can be used to estimate photosynthetic activity and light utilization efficiency [24]. Another possibility to determine the photosynthetic activity is using a Mini-PAM-II from Walz (Effeltrich, Germany).

A study with thyme (*Thymus vulgaris* L.), marjoram (*Origanum majorana* L.), oregano (*Origanum vulgare* L.), lemon balm (*Melissa officinalis* L.), and peppermint (*Mentha piperita* L.) under 50% shading showed, except from lemon balm, a higher yield of total extractive substances (TESs) due to shading. However, the phenol and flavonoid contents of lemon balm increased as a result of shading [17]. There are also studies on the influence of black, red and blue shading nets with 50% photosynthetically active radiation (PAR) reduction on *Mentha arvensis*. Plant growth, EO content and composition were influenced by shading. Both biomass and EO yield were significantly higher under full sunlight than under shading. The EO composition was not significantly affected, but the compound germacrene D was detected only under shading [25].

Therefore, the objective of this study was to investigate the influence of shading (50%) on three different *Mentha* genotypes (*Mentha* × *piperita* ‘Multimentha’, *Mentha* × *piperita* ‘Fränkische Blaue’ and *Mentha rotundifolia* ‘Apfelminze’, which will be called ‘Multimentha’, ‘Fränkische Blaue’ or ‘Fr. Blaue’ and ‘Apfelminze’, respectively, in this paper). These *Mentha* genotypes were cultivated under field conditions for two years (2022–2023). The aim of the experiment was to positively influence the EO composition through green shading. The following hypotheses were made. First, shading reduces abiotic stress and thus leads to a higher yield in biomass. Second, genotypic differences in quantity and quality of EOs are expected. Third, shading leads to an EO composition with a higher proportion of desired compounds such as menthol.

## 2. Results

### 2.1. Temperature and Relative Humidity

**Figure 1:**
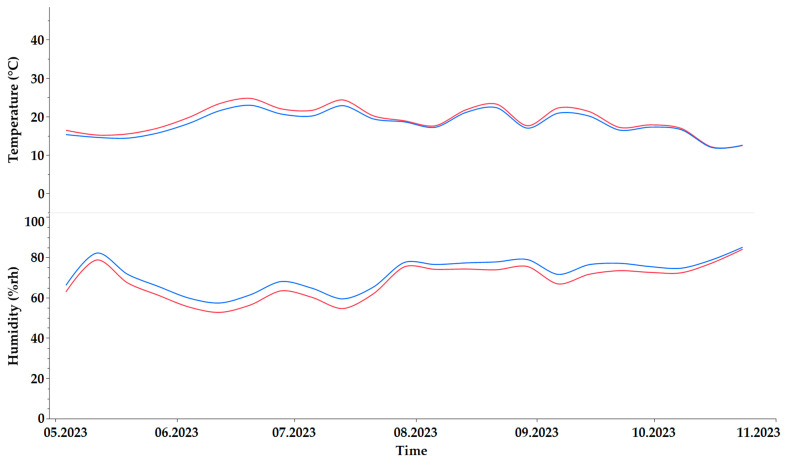
Temperature (°C) and humidity (%rh) under control conditions (red) and shading (blue) in 2023.

The influence on air temperature and relative humidity of shading compared to the control, exemplified for the year 2023, is shown in Figure 1. Statistical analysis revealed a small but significant difference between the control and the shading, probably due to the large sample size of measurements (n = 24,906). On average, the control had a temperature of 19.4 °C and a humidity of 68.0%rh. The shaded samples showed an average temperature of 18.4 °C and a humidity of 71.7%rh. The minimum temperature was −1.5 °C for the control and −0.5 °C for the shading; the maximum was 46 °C for the control and 39.5 °C for the shading. The minimum humidity was 16.5%rh for the control and 23%rh for the shading; the maximum was 97.5%rh for the control and 95.5%rh for the shading. Accordingly, the range of the control in terms of temperature was 47.5 °C and that of the shading 40 °C, while the ranges for humidity were 81%rh (control) and 72.5%rh (shaded). It can therefore be concluded that the shading had only a minor effect on the microclimate.

### 2.2. Plant Height

**Figure 2:**
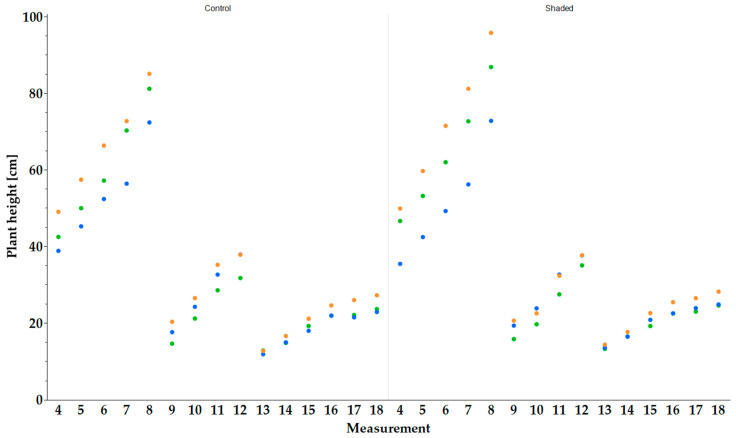
Plant height (cm) of three genotypes, ‘Multimentha’ (orange), ‘Fränkische Blaue’ (blue) and ‘Apfelminze’ (green), under control (**left**) and shaded (**right**) conditions for three harvests (first harvest on 29 June 2022, second on 9 August 2022, third on 11 October 2022). Plant height was measured using 20 plants per genotype and reported as mean value.

In 2022, ‘Multimentha’ reached the highest plant height under both control and shaded conditions in all measurements (Figure 2). The highest plant heights were reached before the first harvest (29 June 2022 (date); 180th day of the year (DOY)) of the genotype ‘Multimentha’ under shaded conditions with up to 95.8 ± 6.3 cm and under control conditions with 85.1 ± 8.2 cm. The second largest was ‘Apfelminze’ with 86.8 ± 8.5 cm (shaded) and 81.2 ± 9.3 cm (control) and then ‘Fränkische Blaue’ with 72.8 ± 7.4 cm (shaded) and 72.4 ± 7.1 cm (control). At the second harvest (9 August 2022; 220), the peppermints ‘Multimentha’ and ‘Fränkische Blaue’ showed higher values in height than ‘Apfelminze’ under shaded conditions (‘Multimentha’ with 37.7 ± 4.7 cm; ‘Fränkische Blaue’ with 37.6 ± 6.3 cm) and control conditions (‘Multimentha’ with 37.8 ± 4.9 cm; ‘Fränkische Blaue’ with 37.9 ± 5.4 cm). ‘Apfelminze’ gained a height of 35.0 ± 3.2 cm (shaded) and 31.7 ± 6.6 cm (control). At the time of the third harvest (11 October 2022; 284), ‘Multimentha’ had the highest plant height in both treatments with 28.2 ± 2.4 cm (shaded) and 27.2 ± 2.1 cm (control). Under shaded conditions, ‘Fränkische Blaue’ yielded 24.8 ± 2.0 cm and ‘Apfelminze’ 24.6 ± 1.7 cm. Under control conditions, ‘Apfelminze’ reached the second highest height with 23.6 ± 3.0 cm and ‘Fränkische Blaue’ with 22.9 ± 2.3 cm. In all three harvests of 2022, the genotypes showed higher plant heights under shaded conditions than under control conditions, but the difference was not significant.

**Figure 3:**
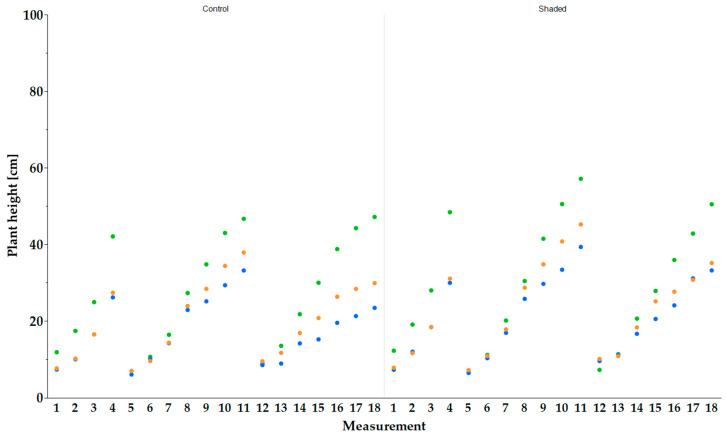
The plant height (cm) of the three genotypes, ‘Multimentha’ (orange), ‘Fränkische Blaue’ (blue) and ‘Apfelminze’ (green), under control conditions (**left**) and shaded (**right**) conditions for three harvests (first harvest on 10 May 2023, second on 20 July 2023, third on 21 September 2023). Plant height was measured using 20 plants per genotype and reported as the mean value.

In 2023, ‘Apfelminze’ reached a significantly higher plant height than the peppermints ‘Multimentha’ (shaded: 31.0 ± 4.2 cm; control: 27.4 ± 4.7 cm) and ‘Fränkische Blaue’ (shaded: 29.9 ± 5.6 cm; control: 26.1 ± 4.7 cm) at the first harvest (10 May 2023; 130) under both shaded (48.4 ± 6.4 cm) and control (42.1 ± 5.6 cm) conditions (Figure 3). At the second harvest (20 July 2023; 201), ‘Apfelminze’ (57.1 ± 10.7 cm) achieved under shaded conditions a significantly higher value than ‘Multimentha’ with 45.2 ± 11.0 cm and ‘Fränkische Blaue’ with 39.3 ± 5.8 cm. Under control conditions, ‘Apfelminze’ also showed the highest value (46.7 ± 10.3 cm) but not significantly higher than ‘Multimentha’ with 37.9 ± 6.5 cm and ‘Fränkische Blaue’ with 33.2 ± 9.9 cm. At the third and final harvest in 2023 (21 September 2023; 264), ‘Apfelminze’ again reached a significantly higher plant height under both conditions (shaded: 50.5 ± 11.5 cm; control: 47.2 ± 12.4 cm) than ‘Multimentha’ (shaded: 35.1 ± 8.7 cm; control: 29.9 ± 7.5 cm) and ‘Fränkische Blaue’ (shaded: 33.2 ± 4.8 cm; control: 23.4 ± 8.1 cm).

### 2.3. Biomass

Biomass accumulation of the three genotypes and two treatments over the year 2022 is shown in Table 1. All three genotypes showed higher biomass accumulation under control conditions compared to shading conditions in 2022, with one exception (second harvest: fresh matter (FM) of ‘Fränkische Blaue’). ‘Multimentha’ had the highest biomass accumulation, independent of treatment, with, e.g., 2307.3 ± 182.5 g/m^2^ FM (control), at the time of the first harvest (29 June 2022; 180). ‘Fränkische Blaue’ showed the second highest biomass values and ‘Apfelminze’ the third highest, although these did not differ as significantly from each other as from ‘Multimentha’. In the second harvest (9 August 2022; 220), ‘Multimentha’ again showed the highest FM (1195.7 ± 339.5 g/m^2^) and dry matter (DM) (305.6 ± 96.5 g/m^2^) under control conditions. Unlike the first harvest, ‘Fränkische Blaue’ showed under shading the second highest FM with 1109.7 ± 305.6 g/m^2^ at the second harvest. At the time of the third harvest (11 October 2022; 284), ‘Fränkische Blaue’ had the highest FM under control conditions with 1030.5 ± 70.2 g/m^2^ and the highest DM with 221.1 ± 16.3 g/m^2^. While there were significant differences between the genotypes for FM and DM, the dry substance (DS) showed no significant differences between the genotypes.

In 2023, ‘Apfelminze’ accumulated the highest biomass under control conditions (Table 2). At the first harvest (10 May 2023; 130), ‘Apfelminze’ had significantly higher FM and DM than the genotypes ‘Multimentha’ and ‘Fränkische Blaue’. In the second harvest (20 July 2023; 201), ‘Apfelminze’ had a slightly higher FM under shaded conditions (1123.2 ± 90.6 g/m^2^) than under control conditions (1099.3 ± 124.6 g/m^2^). ‘Multimentha’ yielded the second highest FM. For DM, however, ‘Multimentha’ showed the highest value with 285.7 ± 69.5 g/m^2^ under control conditions, followed by ‘Apfelminze’ under control conditions (274.0 ± 28.8 g/m^2^) and under shading (233.5 ± 19.9 g/m^2^). In the third harvest (21 September 2023; 264), ‘Apfelminze’ again reached the highest values under control conditions (FM: 1195.3 ± 302.1 g/m^2^; DM: 238.9 ± 62.7 g/m^2^). Regarding FM, the second highest value was also ‘Apfelminze’ with 924.3 ± 213.4 g/m^2^ under shaded conditions and for the DM, ‘Multimentha’ showed the second highest value under control conditions with 218.6 ± 42.5 g/m^2^. For DS, all three genotypes had higher values under control conditions compared to shading.

### 2.4. Vegetation Indices

**Figure 4:**
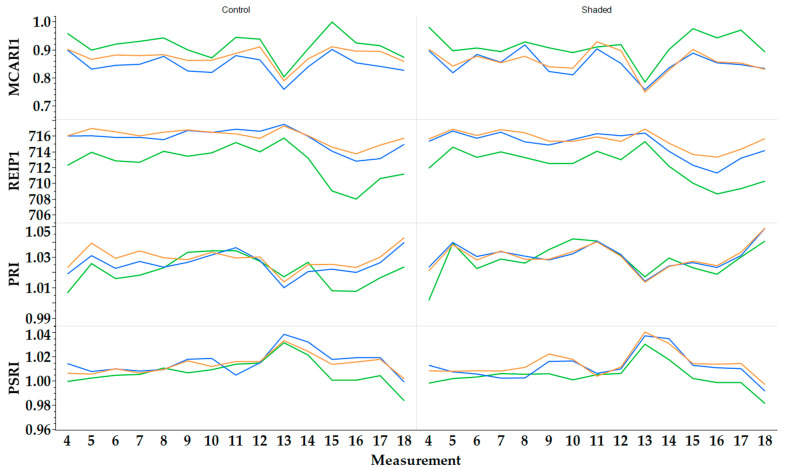
Vegetation indices MCARI1, REIP1, PRI and PSRI of the three genotypes ‘Multimentha’ (orange), ‘Fränkische Blaue’ (blue) and ‘Apfelminze’ (green) under control conditions (**left**) and shading (**right**) in 2022.

In 2022, ‘Apfelminze’ showed the highest mean Modified Chlorophyll Absorption Reflectance Index 1 (MCARI1) under control conditions with 0.915 ± 0.056 (Figure 4). The third and fourth highest were for ‘Multimentha’ (control: 0.876 ± 0.053; shaded: 0.858 ± 0.055). ‘Fränkische Blaue’ had the lowest (control: 0.847 ± 0.049; shaded: 0.851 ± 0.052). For the Red Edge Inflection Point 1 (REIP1), ‘Multimentha’ and ‘Fränkische Blaue’ showed a higher REIP1 than ‘Apfelminze’, irrespective of the treatment. The highest REIP1 was achieved by ‘Multimentha’ (control: 715.9 ± 1.5; shaded: 715.5 ± 1.7). ‘Apfelminze’ reached 712.7 ± 2.6 (control) and 712.3 ± 2.3. In the case of the Photochemical Reflectance Index (PRI), all three genotypes showed similar values. Under shaded conditions, however, the PRIs were slightly higher on average (1.028–1.030) than under control conditions (1.021–1.029). For the Plant Senescence Reflectance Index (PSRI), the genotypes showed slightly higher values under control conditions (‘Multimentha’: 1.014 ± 0.010; ‘Fränkische Blaue’: 1.015 ± 0.012; ‘Apfelminze’: 1.007 ± 0.012) than under shaded conditions (‘Multimentha’: 1.014 ± 0.012; ‘Fränkische Blaue’: 1.012 ± 0.014; ‘Apfelminze’: 1.004 ± 0.011).

**Figure 5:**
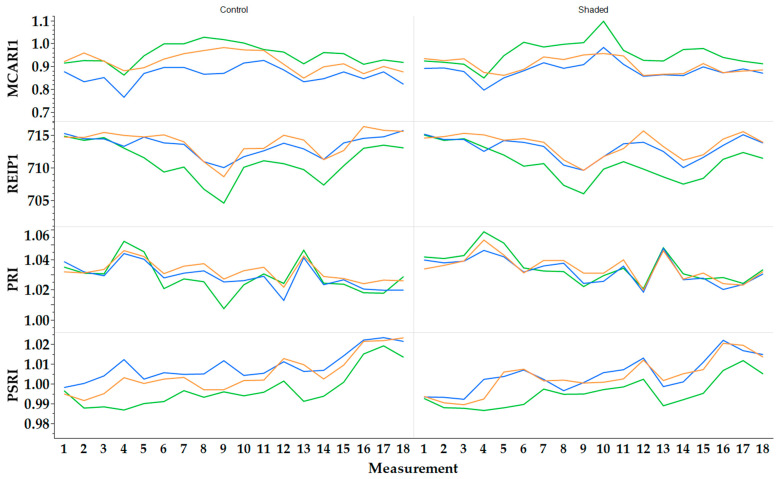
Vegetation indices MCARI1, REIP1, PRI and PSRI of the three genotypes ‘Multimentha’ (orange), ‘Fränkische Blaue’ (blue) and ‘Apfelminze’ (green) under control conditions (**left**) and shading (**right**) in 2023.

As in 2022, ‘Apfelminze’ had the highest MCARI1 in 2023 (Figure 5), both under control conditions (0.952 ± 0.057) and under shaded conditions (0.954 ± 0.071). Then, ‘Multimentha’ (control: 0.920 ± 0.061; shaded: 0.904 ± 0.053) and ‘Fränkische Blaue’ (control: 0.863 ± 0.055; shaded: 0.883 ± 0.062) followed. Under both conditions, ‘Multimentha’ had the highest REIP1 (control: 713.9 ± 2.7; shaded: 713.5 ± 2.5), ‘Fränkische Blaue’ the second highest (control: 713.4 ± 2.8; shaded: 712.9 ± 2.4) and ‘Apfelminze’ the lowest (control: 710.9 ± 3.5; shaded: 710.7 ± 3.2). For PRI, the genotypes under shaded conditions (1.033–1.035) achieved slightly higher values than under control conditions (1.028–1.032). The PSRI values for ‘Apfelminze’ (control: 0.997 ± 0.011; shaded: 0.995 ± 0.009) were slightly lower than for ‘Multimentha’ (control: 1.005 ± 0.011; shaded: 1.004 ± 0.011) or ‘Fränkische Blaue’ (control: 1.009 ± 0.010; shaded: 1.004 ± 0.011).

### 2.5. Electron Transport Rate

**Figure 6:**
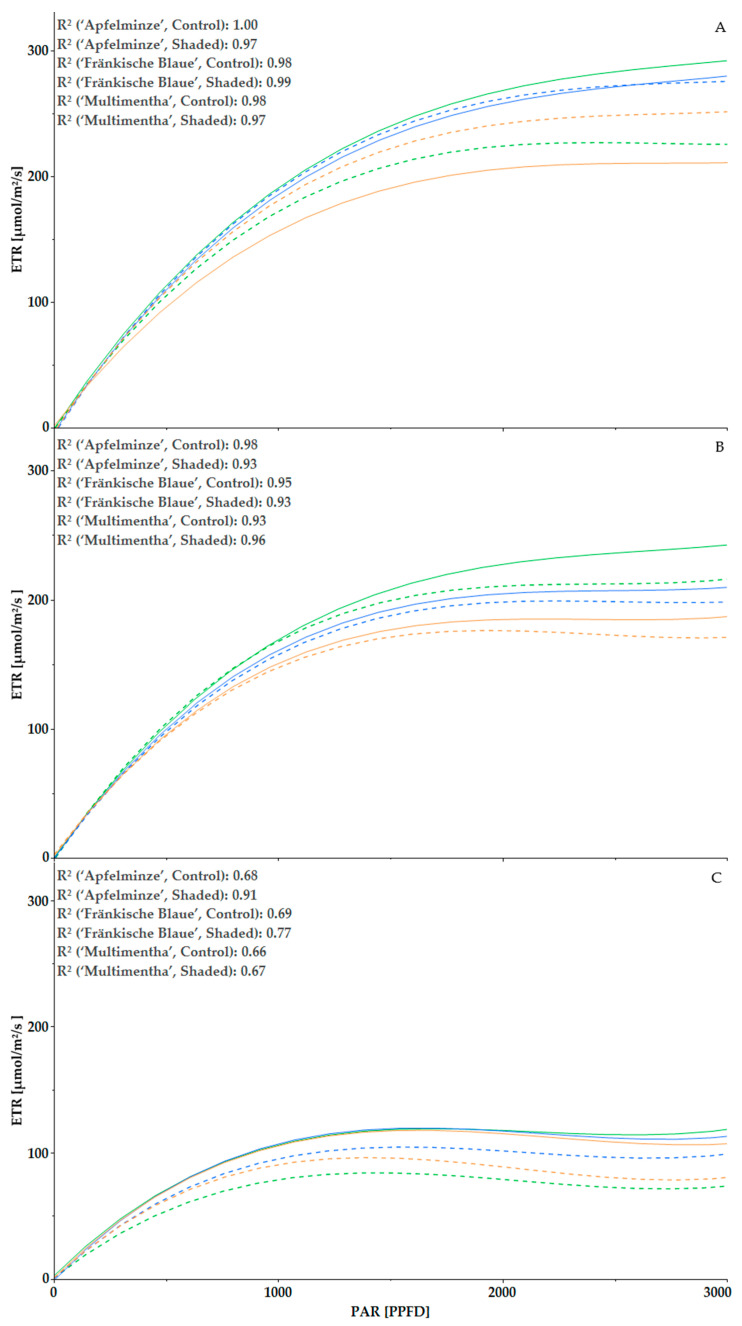
Regression curve of photosynthetic activity of three genotypes, ‘Multimentha’ (orange), ‘Fränkische Blaue’ (blue) and ‘Apfelminze’ (green), under control conditions (solid line) and shading (dashed line) at time of ninth measurement (20 July 2022; (**A**)), twelfth measurement (9 August 2022; (**B**)) and sixteenth measurement (29 September 2022; (**C**)), including coefficient of determination R^2^.

**Figure 7:**
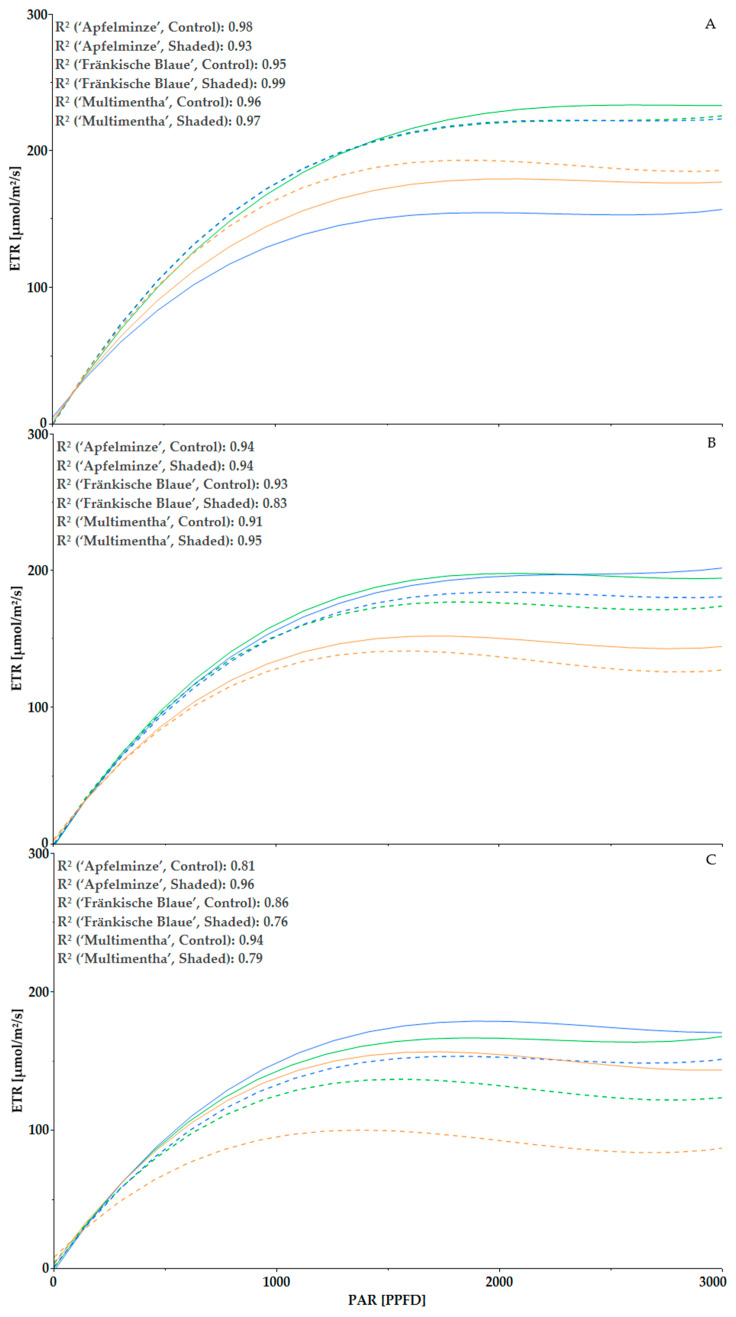
A regression curve of the photosynthetic activity of the three genotypes, ‘Multimentha’ (orange), ‘Fränkische Blaue’ (blue) and ‘Apfelminze’ (green), under control conditions (solid line) and shading (dashed line) at the time of the seventh measurement (21 June 2023; (**A**)), the fourteenth measurement (23 August 2023; (**B**)) and the seventeenth measurement (14 September 2023; (**C**)), including the coefficient of determination R^2^.

The photosynthetic activity, measured via the electron transport rate (ETR), of the six varieties for the year 2022 (Figure 6) and for the year 2023 (Figure 7) is shown by three measurement dates for each year. At the ninth measurement in 2022, the shaded varieties still showed a higher ETR (in µmol electrons/m^2^/s) than under control conditions. The exception was ‘Apfelminze’, which had the highest ETR under control conditions (191.0 µmol/m^2^/s at a PAR (photosynthetically active radiation) of 1000 µmol/m^2^/s PPFD (photosynthetically active photon flux density)). The lowest was ‘Multimentha’, with an ETR of 156.6 µmol/m^2^/s at 1000 PPFD under control conditions. The ETR changed over the course of the year. At the twelfth measurement, ‘Apfelminze’ had the highest ETR under both conditions, followed by ‘Fränkische Blaue’ and ‘Multimentha’. The control condition showed a higher value than the shaded condition for all three genotypes. ‘Apfelminze’ (control) had an ETR of 169.1 µmol/m^2^/s at 1000 PPFD and ‘Multimentha’ (shaded) of 147.6 µmol/m^2^/s. Towards the end of the measurement period at the sixteenth measurement, all three genotypes achieved very similar ETR values under control conditions (‘Apfelminze’: 106.4 µmol/m^2^/s, ‘Multimentha’: 105.9 µmol/m^2^/s and ‘Fränkische Blaue’: 107.3 µmol/m^2^/s at 1000 PPFD) and thus stood out from the shaded genotypes. Under shading, ‘Fränkische Blaue’ achieved an ETR of 95.5 µmol/m^2^/s, ‘Multimentha’ of 90.8 µmol/m^2^/s and ‘Apfelminze’ of 78.8 µmol/m^2^/s at a PAR of 1000 PPFD. After reaching 1000 PPFD, the curves rose more flatly up to around 2000 PPFD, after which saturation set in.

In the beginning of 2023, ‘Apfelminze’ (shaded) and ‘Fränkische Blaue’ (shaded) initially showed the highest ETR with 176.0 µmol/m^2^/s (‘Apfelminze’) and 175.8 µmol/m^2^/s (‘Fränkische Blaue’) at a PAR of 1000 PPFD in the seventh measurement, followed by ‘Apfelminze’ (control) with 172.1 µmol/m^2^/s, ‘Multimentha’ (shaded) with 164.4 µmol/m^2^/s, ‘Multimentha’ (control) with 147.7 µmol/m^2^/s and ‘Fränkische Blaue’ (control) with 131.8 µmol/m^2^/s (Figure 7). As in the previous year, the values changed over the course of time, with the shaded plants having a lower ETR than those under control conditions. At the fourteenth measurement, ‘Apfelminze’ with 160.3 µmol/m^2^/s and ‘Fränkische Blaue’ with 156.8 µmol/m^2^/s showed the two highest ETR values under control conditions at a PAR of 1000 PPFD. Subsequently, ‘Apfelminze’ (152.6 µmol/m^2^/s) and ‘Fränkische Blaue’ (152.2 µmol/m^2^/s) under shaded conditions followed. ‘Multimentha’ had the lowest ETR (control: 134.5 µmol/m^2^/s and shaded: 128.2 µmol/m^2^/s). In the seventeenth measurement, all three genotypes again showed higher values under control conditions than under shaded conditions. These were ‘Fränkische Blaue’ with 149.1 µmol/m^2^/s, ‘Apfelminze’ with 143.1 µmol/m^2^/s and ‘Multimentha’ with 137.7 µmol/m^2^/s at 1000 PPFD (control) and under shaded conditions, ‘Fränkische Blaue’ with 132.5 µmol/m^2^/s, ‘Apfelminze’ with 125.0 µmol/m^2^/s and ‘Multimentha’ with 95.2 µmol/m^2^/s.

### 2.6. Essential Oil Content

**Figure 8:**
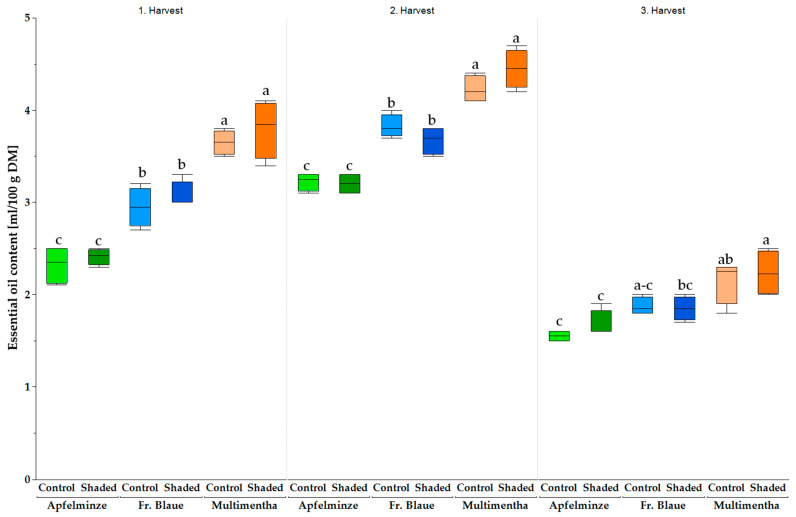
The essential oil content (ml/100 g DM) of the three genotypes, ‘Multimentha’ (orange), ‘Fränkische Blaue’ (blue) and ‘Apfelminze’ (green), under control conditions (brighter color) and shading (darker color) over three harvests (the first harvest on 29 June 2022, second on 9 August 2022, third on 11 October 2022). Significant differences are indicated by letters after ANOVA and Tukey HSD (*p* ≤ 0.05).

EO content varied significantly between genotypes (Figure 8). The EO content was the highest in the second harvest of 2022. ‘Multimentha’ showed the highest EO contents under shaded conditions in all three harvests (first harvest: 3.8 ± 0.3 mL/100 g DM; second harvest: 4.5 ± 0.2 mL/100 g DM; third harvest: 2.2 ± 0.2 mL/100 g DM) (Figure 8). The second highest values were reached by ‘Multimentha’ under control conditions (first harvest: 3.7 ± 0.1 mL/100 g DM; second harvest: 4.2 ± 0.2 mL/100 g DM; third harvest: 2.1 ± 0.2 mL/100 g DM), while ‘Fränkische Blaue’ and ‘Apfelminze’ had the lowest EO contents under control conditions (first harvest: 2.3 ± 0.2 mL/100 g DM; second harvest: 3.2 ± 0.1 mL/100 g DM; third harvest: 1.6 ± 0.1 mL/100 g DM).

**Figure 9:**
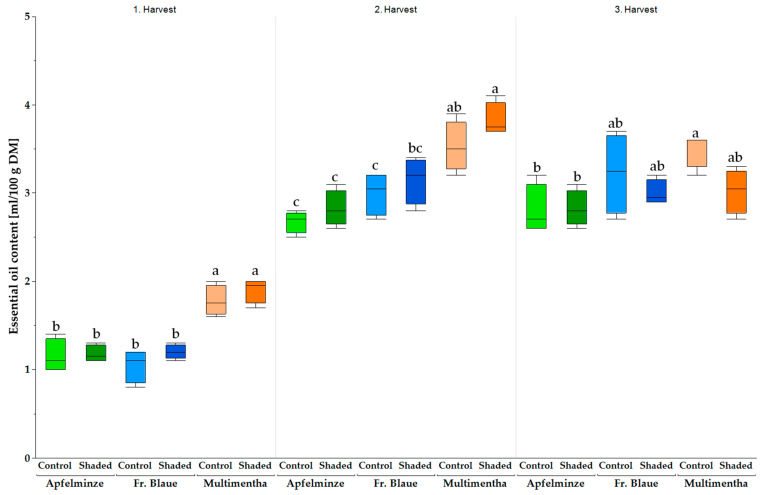
The essential oil content (ml/100 g DM) of the three genotypes, ‘Multimentha’ (orange), ‘Fränkische Blaue’ (blue) and ‘Apfelminze’ (green), under control conditions (brighter color) and shading (darker color) over three harvests (the first harvest on 10 May 2023, second on 20 July 2023, third on 21 September 2023). Significant differences are indicated by letters after ANOVA and Tukey HSD (*p* ≤ 0.05).

Significant differences in the EO content between the genotypes were also noticeable in 2023. However, unlike in 2022, the second and third harvests were more productive than the first (Figure 9). ‘Multimentha’ again showed the highest values under shaded conditions (first harvest: 1.9 ± 0.1 mL/100 g DM; second harvest: 3.8 ± 0.2 mL/100 g DM; third harvest: 3.0 ± 0.3 mL/100 g DM). ‘Apfelminze’ also had the lowest EO content under control conditions (first harvest: 1.2 ± 0.2 mL/100 g DM; second harvest: 2.7 ± 0.1 mL/100 g DM; third harvest: 2.8 ± 0.3 mL/100 g DM).

### 2.7. Essential Oil Composition

EO composition varied significantly between the three genotypes. ‘Fränkische Blaue’ had the highest number in compounds with 7–10 depending on the year, harvest and treatment. ‘Multimentha’ had 7–9 and ‘Apfelminze’ 5–7. Across all years, harvests, and treatments, the main compound of ‘Apfelminze’ was carvone and that of the peppermints (‘Multimentha’ and ‘Fränkische Blaue’) was *p*-menthone followed by menthol isomer B (Table 3, Table 4 and Table 5). Pulegone was not detected in the first harvest in either year, 1,6-dihydrocarveol, menthyl acetate and β-bourbonene were not detected in the second harvest and in the third harvest, β-bourbonene was again not detected.

In ‘Apfelminze’, eucalyptol/limonene were more present in both 2022 and 2023 under control conditions (e.g., 2023: 11.84 ± 0.59%) than under shaded conditions (e.g., 2023: 8.76 ± 5.15%). Dihydrocarvone showed higher values in both years under control conditions (e.g., 2023: 13.41 ± 1.76%) than under shaded conditions (e.g., 2023: 10.45 ± 3.16%). However, the main component carvone was found in slightly higher quantities under shaded conditions (e.g., 2023: 68.54 ± 7.08%) than under control conditions (e.g., 2023: 62.25 ± 3.25%).

‘Fränkische Blaue’ had a slightly higher *p*-menthone content under control conditions (42.33 ± 1.70%) than under shaded conditions (41.79 ± 4.13%) in 2022, but a higher *p*-menthone content under shaded conditions (44.77 ± 6.02%) than under control conditions (38.33 ± 12.47%) in 2023. However, the menthol isomer B content behaves more evenly with higher values under control conditions in both years (e.g., 2023: 38.99 ± 5.80%) instead of shaded conditions (e.g., 2023: 33.32 ± 5.68%).

The *p*-menthone content of ‘Multimentha’ was in line with ‘Fränkische Blaue’ and had a higher content of 54.03 ± 2.22% under control conditions than under shading (52.69 ± 4.04%) in 2022. In 2023, however, it was slightly higher under shaded conditions (57.82 ± 2.71%) than under control conditions (57.54 ± 2.22%). In 2023, ‘Multimentha’ was the only genotype that contained menthofuran in the first harvest with 1.13 ± 0.12% under shaded conditions.

As in the first harvest, ‘Apfelminze’ in the second harvest had a higher eucalyptol/limonene value under control conditions (Table 4). However, carvone was present in higher quantities under shaded conditions (e.g., 75.11 ± 1.32%, 2023) than under control conditions (e.g., 73.46 ± 1.08%, 2023).

In both years, ‘Fränkische Blaue’ with 55.22 ± 2.16% (2022) and 49.56 ± 3.18% (2023) and ‘Multimentha’ with 67.35 ± 1.03% (2022) and 65.36 ± 2.13% (2023) showed higher *p*-menthone values under shaded conditions than under control conditions. However, the menthol isomer B values were lower under shaded conditions in both years. In contrast to the first harvest, menthofuran and pulegone were only present in the peppermints (‘Fränkische Blaue’ and ‘Multimentha’) in the second harvest. Except for ‘Fränkische Blaue’ in 2022 (1.58 ± 0.16%, shaded; 2.12 ± 0.15% control), the shaded conditions showed lower menthofuran values than the control, for example, ‘Multimentha’ in 2023 under control conditions 2.81 ± 0.26% and under shaded conditions 2.12 ± 0.25%. In 2022, pulegone was still detected under control and shaded conditions, but with lower levels under shaded conditions (‘Fränkische Blaue’: 1.03 ± 0.40%; ‘Multimentha’: 2.32 ± 0.39%) than under control conditions (‘Fränkische Blaue’: 1.59 ± 0.15%; ‘Multimentha’: 2.67 ± 0.39%). In 2023, pulegone was only detected under control conditions with 1.09 ± 0.34% in ‘Fränkische Blaue’ and 1.83 ± 0.28% in ‘Multimentha’.

In the third harvest (Table 5), as in the first and second harvests in both years, ‘Apfelminze’ showed a higher eucalyptol/limonene content under control conditions (12.88 ± 0.35%, 2022; 13.32 ± 0.54%, 2023) than under shaded conditions (11.55 ± 0.55%, 2022; 10.06 ± 1.79%, 2023). Carvone also showed a higher value under control conditions, in contrast to the first two harvests where higher values were recorded under shaded conditions. Under control conditions, carvone values were 68.20 ± 3.59% (2023) and under shaded conditions, they were 67.84 ± 4.25% (2023). In contrast to the control conditions (e.g., 10.35 ± 3.47% (2023)), dihydrocarvone showed higher values under shaded conditions (e.g., 13.50 ± 4.64% (2023)).

As in the second harvest, the peppermints ‘Fränkische Blaue’ and ‘Multimentha’ had a higher *p*-menthone value under shaded conditions in the third harvest in both years: ‘Fränkische Blaue’ with 44.71 ± 1.27% (2022) and 46.84 ± 2.81% (2023) and ‘Multimentha’ with 60.27 ± 3.12% (2022) and 60.07 ± 1.00% (2023). Meanwhile, under control conditions, ‘Fränkische Blaue’ had 39.85 ± 0.94% (2022) and 45.56 ± 2.93% (2023) and ‘Multimentha’ 55.91 ± 2.09% (2022) and 58.35 ± 1.26% (2023). However, the menthol isomer B contents were lower under shaded conditions than under control conditions. Menthofuran and pulegone were also detected in the third harvest, but only in 2023. Menthofuran was detected in ‘Fränkische Blaue’ with 3.38 ± 0.42% (control) and 3.26 ± 0.84% (shaded) and in ‘Multimentha’ with 5.07 ± 0.61% (control) and 5.88 ± 0.84% (shaded). Pulegone was only present in ‘Multimentha’ with 1.20 ± 0.11% (control) and 1.50 ± 0.20% (shaded).

## 3. Discussion

The first hypothesis stated that shading reduces abiotic stress and thus leads to a higher biomass yield. The vegetation index PSRI confirms a slight reduction in stress, though not significant, as PSRI was lower under shaded conditions in 2022 and 2023 for all three genotypes. Plant height was higher under shaded conditions in comparison to control conditions in both years (e.g., ‘Apfelminze’ at the second harvest in 2023: 57.1 ± 10.7 cm, shaded; 46.7 ± 10.3 cm, control). However, the biomass yield showed an opposite trend compared to the plant height in 2022 and was lower under shaded conditions than under control conditions for all genotypes. In 2023, there was no clear trend for changes in FM and DM under shading or control conditions. This is supported by the results of Deraman et al., where the highest plant height was found in a *Mentha arvensis* with a 50% reduction in light but the highest biomass accumulation in the unshaded control [26]. If achieving high biomass yields is a primary goal of cultivating *Mentha*, such as for tea, shading is not necessarily beneficial unless further examination of leaf size and the leaf/stem ratio reveals that shading promotes larger and more leaves. Here, the leaf/stem ratio could be an indicator as well. Shading also does not ensure that the photosynthetic activity of the mint adapts to the light conditions. This was shown by the measurement results of the ETR, determined by the Mini PAM. The ETR was higher under non-shaded conditions than under control conditions, especially in the high PAR range (1500–3000 PPFD). However, this is more important for regions with a higher average PAR than Central Europe. During the experiment period, for example, the average PAR in 2022 was 1430 ± 434 PPFD under control conditions and in 2023 it was 1331 ± 613 PPFD.

If the focus of mint cultivation is on EO extraction, EO content is the most important factor that needs to be considered. Furthermore, EO composition is of major importance. We hypothesized that there will be genotypic differences in EO content and composition as a consequence of shading. For this reason, the three *Mentha* genotypes (*Mentha* × *piperita* ‘Multimentha’, *Mentha* × *piperita* ‘Fränkische Blaue’ and *Mentha rotundifolia* ‘Apfelminze’) were selected so that both inter- and intra-species comparisons could be made. The differences in EO content between the harvests of one year are mostly due to the various seasonal influences and are therefore not evaluated further. However, there were significant differences in both EO content and composition between the genotype ‘Apfelminze’ and the peppermints (‘Multimentha’ and ‘Fränkische Blaue’), and also slight differences between the two peppermints. ‘Multimentha’ showed the highest EO content compared to the other mints over both years, all three harvests within each year, and under both control and shaded conditions (e.g., second harvest in 2022: 4.2 mL/100 g DM, control; 4.5 mL/100 g DM, shaded). In contrast, ‘Apfelminze’ reached significantly lower values (e.g., second harvest in 2022: 1.6 mL/100 g DM, control; 1.7 mL/100 g DM, shaded). This difference was to be expected, as ‘Apfelminze’ is used as a tea mint and ‘Multimentha’ for EO extraction. However, significant intra-species differences were also found between the two peppermints (e.g., first harvest in 2023: 1.05 ± 0.2 mL/100 g DM (‘Fränkische Blaue’, control); 1.8 ± 0.2 mL/100 g DM (‘Multimentha’, control) and 1.2 ± 0.1 mL/100 g DM (‘Fränkische Blaue’, shaded); 1.9 ± 0.1 mL/100 g DM (‘Multimentha’, shaded)). Therefore, a selection for cultivation should not only be made at the species level but also at the genotype level [27].

However, different chemotypes can also manifest themselves within a genotype; for example, *Mentha longifolia* has the limonene-oxo and γ-terpinene pathways and can thus have different EO compositions [28]. Different ecotypes may also develop which are only adapted to certain environments. The EO content under shading is unchanged or slightly higher than under control conditions. The only exception to this is the third harvest in 2023, presumably due to a change in weather with significantly lower irradiation. This means that green shading does not increase the yield of the EO under Central European climatic conditions, but the quality of the EO is also crucial for generating value and the genotypes also differed in their EO composition.

While ‘Apfelminze’ had carvone as its main compound, the peppermints ‘Multimentha’ and ‘Fränkische Blaue’ had *p*-menthone. Yu et al. (2018) also found menthone to be the main compound in *Mentha* × *piperita* Linn. [29]. It was hypothesized that green shading leads to an EO composition with a higher proportion of desired compounds such as menthol and carvone [30]. Menthol is the most common compound in peppermint EO and mainly determines its value [31]. In addition to the desired compounds, there are also undesirable ones such as pulegone and menthofuran, which should ideally not be present or should be below the limit value (≤1% is graded as safe [10]). Shading did not lead to a significant change in the EO composition; genotypic and seasonal differences had a stronger influence. However, it was noticeable that the concentration of pulegone and menthofuran increased from the first to the second harvest and then decreased again from the second to the third harvest. It might be concluded that UV stress, which has an increased effect on plants in summer, leads to the formation of these compounds [32], and that shading may contribute with slightly higher light transmission to better EO quality in latitudes or altitudes with higher UV radiation.

In summary, it can be said that 50% green shading does not lead to significant changes in EO content or composition under the climatic conditions in Central Europe. However, this trial should be repeated under climatic and soil conditions different from the central European climate and a silty clay soil, and with different shading values. In addition, other shading net colors should also be considered, as this change in the incident light spectrum could have an impact on biomass or EO. Different light spectra have already proven to have effects on plants in indoor cultivation. For example, increasing the red component of the light spectrum provided a higher DM and a higher blue component of the light spectrum increased terpenoid concentrations, e.g., eugenol, in Sweet Basil (*Ocimum basilicum*) leaves [33], and this can possibly be transferred to outdoor cultivation. A broader selection of genotypes would also be of interest, as each genotype reacts differently to different environmental influences. It would also be interesting to investigate the effect of changes in the light spectrum on the phytohormones, as light is the main signal for hormone action. The hormones can in turn have positive and negative effects on photosynthesis and the adaptive response of plants [34].

In conclusion, it can be said that extended trials under other climatic conditions, different shading values and colors, and trials with a larger selection of genotypes is necessary.

## 4. Materials and Methods

### 4.1. Plant Material and Cultivation

Three *Mentha* genotypes were selected for the field experiment: *Mentha* × *piperita* ‘Multimentha’, *Mentha* × *piperita* ‘Fränkische Blaue’ and *Mentha rotundifolia* ‘Apfelminze’, which have been called ‘Multimentha’, ‘Fränkische Blaue’ or ‘Fr. Blaue’ and ‘Apfelminze’, respectively, in this paper. These genotypes are also grown commercially in Germany. The plant material for this experiment was propagated via cuttings from plants that were cultivated in a greenhouse at Campus Klein-Altendorf (University of Bonn, Rheinbach, Germany) in 2021. The parent material for these plants originated from a genotype assortment that was installed at Campus Klein-Altendorf in 2016 and cultivated under field conditions since then. The cuttings were made on 23 July 2021 and were grown under greenhouse conditions (16 h light/8 h dark) and were watered every 48 h via an ebb–flow system. The cuttings were trimmed back on 11 August 2021 and planted out in the field on 14 September 2021 using a mechanical three-row planting machine. This resulted in 24 plots measuring three by five meters with three-meter-wide intermediate paths to avoid hybridization. There were six rows of mint and 60 plants (4 plants/m^2^) per plot (45 cm row width). Using three genotypes (‘Multimentha’, ‘Fränkische Blaue’ and ‘Apfelminze’) and two treatments (control and shading), this results in four replicates per variety and six varieties in total. The experiment was carried out over two years (2022–2023) with three harvests per year. The soil type was a silty clay. Temperature and precipitation were recorded over the course of the experiment (Table 6). The sum of precipitation in 2022 was 493.1 mm and in 2023, it was 644.3 mm. The average temperature in 2022 was 11.6 ± 7.0 °C and in 2023 11.6 ± 6.6 °C.

The shaded plots were provided with 1.2 m high poles and roof battens above them. The green shading net (Hartmann-Brockhaus, Pfaffenhofen-Wagenhofen, Germany) was reversibly attached to this construction so that the net could be moved aside during data collection and harvest. In order to not interfere with the microclimate, the shading net was not completely closed on the sides. Climate data were documented using Omega Datalogger ‘OM-EL-USB-2-PLUS’ (Deckenpfronn, Germany). The rows at the edges still received full sunlight from the side, but the inner rows were completely shaded. According to the manufacturer, the shading performance was 50%. This was verified with UV-A, UV-B and PAR value measurements with a Gigahertz-Optik X12 Optometer (Gigahertz-Optic; Member of the GERGHOF GROUP, Türkenfeld, Germany). UV-A and UV-B radiation was measured in W/m^2^ and PAR in photosynthetic photon flux density (PPFD) in µmol/m^2^/s (Table 7).

### 4.2. Harvest and Data Collection

In both years, 18 measurements (Table 8) were conducted on a weekly basis (2022: May–October; 2023: April–September). In 2022, the shading nets were installed at the fifth measurement date and three harvests were carried out (first harvest in June, second in August, third in October). In 2023, the shading was installed at the first measurement. Three harvests were carried out again (first harvest in May, second in July, third in September). Plant height and the physiological reaction of *Mentha* (20 plants per variant) via hyperspectral measurements and calculation of vegetation indices (VIs) using a PolyPen RP400 (UV-VIS) by Photon Systems Instruments (Drásov, Czech Republic) were measured weekly. The PolyPen has a spectral response range of 380–790 nm. The VIs MCARI1, PRI, PSRI and REIP1 were obtained. With the help of the MCARI1 (Modified Chlorophyll Absorption Reflectance Index 1), the chlorophyll and green coloration can be determined [35]. The PRI (Photochemical Reflectance Index) can be used to estimate photosynthetic activity and light use efficiency of the mints [24]. The PSRI (Plant Senescence Reflectance Index) responds to changes in the ratio of carotenoids to chlorophyll and thus can be used to determine leaf senescence [36]. The REIP1 (Red Edge Inflection Point 1) determines chlorophyll content and draws conclusions about the nitrogen content [37]. Furthermore, the measurements included the identification of the photosynthetic activity using a Mini-PAM-II from Walz (Effeltrich, Germany). For this purpose, a part of the leaf was first darkened for around 30 min using a leaf clip in order to reduce photosynthetic activity to zero. The leaf was then exposed to an internal PAR and the photosynthetic activity was measured until the maximal PAR of around 3590 PPFD was reached. Represented as regression curves, equations are shown in the Supplementary Material (Table S1). In addition, biomass (Fresh matter (FM), Dry matter (DM), Dry substance (DS)) and EO content were determined at each harvest. One square meter of plant material was harvested for the biomass samples. Plant samples were dried for five days at 35 °C (Venticell 707—Eco Line, MMM Group, Planegg, Germany).

### 4.3. Essential Oil Extraction and Analysis

The EOs were extracted by steam distillation according to the methodology of the European Pharmacopoeia [8] using the ‘KOL’ and ‘KOL 2’ apparatuses (behr Labor-Technik GmbH, Düsseldorf, Germany). The analysis of the composition of EO was carried out using an Agilent 7890B gas chromatograph (Agilent Technologies, Palo Alto, CA, USA) equipped with a ZEBRON ZB 1 MS (30 m, 0.25 mm i.d. × 0.1 µm df). Both extractions were carried out as described by Hubert et al. [15].

### 4.4. Statistical Analysis

Data are given as means with standard deviations. Statistical analysis was performed using JMP Pro 17 (SAS Institute GmbH, Heidelberg, Germany). Calculations were made via analysis of variance (ANOVA) with Tukey HSD as a post hoc procedure to determine homogenous subgroups at a *p*-value of *p* ≤ 0.05, indicated by letters.

## 5. Conclusions

- A selection for cultivation should not only be made at the species level but rather at the genotype level because of both inter- and intra-specific differences.
- EO content under shading is slightly higher than under control conditions.
- In latitudes or altitudes with higher UV radiation, shading might contribute to higher EO quality.
- To gain high quantities of mint biomass, shading is not necessarily beneficial unless further examination of the leaf size and leaf–stem ratio reveals that shading promotes larger and more leaves.

## Figures and Tables

**Table 1:** Biomass accumulation (g/m^2^) of the three *Mentha* genotypes (‘Apfelminze’, ‘Fränkische Blaue’, ‘Multimentha’) and two treatments (shaded and control) for the year 2022 and three harvests. Significant differences calculated by ANOVA and Tukey HSD (n = 72, α = 0.05) are indicated by letters (a–d) for fresh matter (FM), dry matter (DM), dry substance (DS) and harvest separately.

Genotype	Treatment	Harvest	FM [g/m^2^]	DM [g/m^2^]	DS [%]
‘Apfelminze’	Shaded	1	1433.6 ± 202.2 c	305.5 ± 47.3 cd	21.3 ± 0.5 cd
‘Apfelminze’	Control	1	1492.2 ± 232.5 c	360.0 ± 57.4 cd	24.1 ± 0.9 ab
‘Fr. Blaue’	Shaded	1	1496.2 ± 301.9 c	295.2 ± 56.2 d	19.8 ± 0.4 d
‘Fr. Blaue’	Control	1	1825.9 ± 188.6 bc	416.7 ± 52.9 bc	22.8 ± 1.4 bc
‘Multimentha’	Shaded	1	2296.1 ± 135.1 ab	494.3 ± 59.9 ab	21.5 ± 1.6 cd
‘Multimentha’	Control	1	2307.3 ± 182.5 a	587.3 ± 39.6 a	25.5 ± 0.8 a
‘Apfelminze’	Shaded	2	628.2 ± 107.8 b	126.4 ± 19.9 b	20.2 ± 1.1 b
‘Apfelminze’	Control	2	645.9 ± 205.6 b	165.6 ± 46.8 ab	25.8 ± 0.8 a
‘Fr. Blaue’	Shaded	2	1109.7 ± 305.6 a	226.9 ± 65.4 ab	20.4 ± 0.8 b
‘Fr. Blaue’	Control	2	974.6 ± 361.6 ab	250.3 ± 90.3 ab	25.8 ± 1.1 a
‘Multimentha’	Shaded	2	890.4 ± 76.9 ab	183.9 ± 11.4 ab	20.7 ± 0.8 b
‘Multimentha’	Control	2	1195.7 ± 339.5 a	305.6 ± 96.5 a	25.4 ± 1.2 a
‘Apfelminze’	Shaded	3	516.0 ± 49.2 d	97.8 ± 12.8 c	18.9 ± 0.9 ab
‘Apfelminze’	Control	3	605.5 ± 56.3 cd	126.8 ± 11.0 bc	20.9 ± 1.3 a
‘Fr. Blaue’	Shaded	3	724.2 ± 151.3 b–d	142.1 ± 27.2 b	19.7 ± 1.2 ab
‘Fr. Blaue’	Control	3	1030.5 ± 70.2 a	221.1 ± 16.3 a	21.5 ± 1.2 a
‘Multimentha’	Shaded	3	791.5 ± 121.2 a–c	143.4 ± 17.8 b	18.2 ± 1.1 b
‘Multimentha’	Control	3	899.7 ± 141.5 ab	185.5 ± 20.7 a	20.7 ± 1.1 ab

**Table 2:** Biomass accumulation (g/m^2^) of the three *Mentha* genotypes (‘Apfelminze’, ‘Fränkische Blaue’, ‘Multimentha’) and two treatments (shaded and control) for the year 2023 and three harvests. Significant differences calculated by ANOVA and Tukey HSD (n = 72, α = 0.05) are indicated by letters (a–c) for fresh matter (FM), dry matter (DM), dry substance (DS) and harvest separately.

Genotype	Treatment	Harvest	FM [g/m^2^]	DM [g/m^2^]	DS [%]
‘Apfelminze’	Shaded	1	1933.4 ± 471.6 a	178.6 ± 43.1 ab	9.2 ± 0.4 c
‘Apfelminze’	Control	1	2200.1 ± 587.8 a	236.5 ± 61.4 a	10.8 ± 0.1 b
‘Fr. Blaue’	Shaded	1	1103.2 ± 199.7 b	121.9 ± 17.3 b	11.1 ± 0.8 b
‘Fr. Blaue’	Control	1	958.7 ± 87.3 b	128.8 ± 13.0 b	13.4 ± 0.4 a
‘Multimentha’	Shaded	1	1150.6 ± 66.5 b	124.6 ± 7.9 b	10.8 ± 0.8 b
‘Multimentha’	Control	1	819.2 ± 262.9 ab	108.9 ± 28.8 b	13.5 ± 0.9 a
‘Apfelminze’	Shaded	2	1123.2 ± 90.6 a	233.5 ± 19.9 ab	20.8 ± 0.7 c
‘Apfelminze’	Control	2	1099.3 ± 124.6 a	274.0 ± 28.8 ab	25.0 ± 1.9 b
‘Fr. Blaue’	Shaded	2	581.4 ± 77.9 c	148.2 ± 21.5 c	25.5 ± 2.3 b
‘Fr. Blaue’	Control	2	659.4 ± 163.3 bc	196.3 ± 42.4 bc	30.0 ± 1.6 a
‘Multimentha’	Shaded	2	799.4 ± 33.7 bc	193.5 ± 2.7 bc	24.2 ± 0.9 b
‘Multimentha’	Control	2	913.7 ± 209.5 a	285.7 ± 69.5 a	31.2 ± 0.8 a
‘Apfelminze’	Shaded	3	924.3 ± 213.4 ab	154.6 ± 43.6 a–c	16.6 ± 0.9 c
‘Apfelminze’	Control	3	1195.3 ± 302.1 a	238.9 ± 62.7 a	20.2 ± 1.1 b
‘Fr. Blaue’	Shaded	3	704.9 ± 161.0 bc	135.9 ± 27.6 bc	19.4 ± 0.9 b
‘Fr. Blaue’	Control	3	408.8 ± 32.9 c	94.6 ± 7.7 c	23.2 ± 1.5 a
‘Multimentha’	Shaded	3	887.9 ± 118.7 ab	165.4 ± 19.9 a–c	18.7 ± 0.4 bc
‘Multimentha’	Control	3	914.3 ± 185.7 ab	218.6 ± 42.5 ab	23.9 ± 0.3 a

**Table 3:** The EO composition (%) of each compound of the first harvest of the years 2022 and 2023 of the three genotypes, ‘Multimentha’ (MM), ‘Fränkische Blaue’ (FB) and ‘Apfelminze’ (AM), under control (C) and shaded (S) conditions. Data are given as the mean with the standard deviation (n = 4). Compound levels below 1% are not listed (indicated by hyphen).

	2022						2023					
Compound	AM C	AM S	FB C	FB S	MM C	MM S	AM C	AM S	FB C	FB S	MM C	MM S
Eucalyptol/ limonene	12.02 ± 0.48	11.25 ± 0.15	3.07 ± 0.28	3.11 ± 0.22	2.41 ± 0.13	2.23 ± 0.07	11.84 ± 0.59	8.76 ± 5.15	-	-	1.16 ± 0.07	-
*p*-Menthone	-	-	42.33 ± 1.70	41.79 ± 4.13	54.03 ± 2.22	52.69 ± 4.04	-	-	38.33 ± 12.47	44.77 ± 6.02	57.54 ± 2.22	57.82 ± 2.71
Isomenthone	-	-	5.99 ± 0.29	5.64 ± 0.42	3.91 ± 0.10	3.82 ± 0.21	-	-	4.86 ± 1.55	5.23 ± 0.58	3.63 ± 0.10	3.85 ± 0.15
Menthofuran	-	-	-	-	-	-	-	-	-	-	-	1.13 ± 0.12
Menthol isomer A	-	-	3.12 ± 0.37	2.92 ± 0.36	4.61 ± 0.44	4.75 ± 0.57	-	-	5.77 ± 0.61	4.85 ± 0.94	9.19 ± 1.63	8.47 ± 0.60
Menthol isomer B	-	-	29.04 ± 0.78	28.38 ± 3.61	23.62 ± 2.43	24.34 ± 3.65	-	-	38.99 ± 5.80	33.32 ± 5.68	20.81 ± 1.52	21.07 ± 1.98
Dihydro- carvone	8.98 ± 1.98	8.27 ± 1.85	-	-	-	-	13.41 ± 1.76	10.45 ± 3.16	-	-	-	-
1,6-Dihydrocarveol	-	-	-	-	-	-	1.91 ± 0.71	1.52 ± 0.52	-	-	-	-
Pulegone	-	-	-	-	-	-	-	-	-	-	-	-
Carvone	67.22 ± 2.43	69.44 ± 2.51	-	-	-	-	62.25 ± 3.25	68.54 ± 7.08	-	-	-	-
Piperitone	-	-	1.46 ± 0.13	1.57 ± 0.08	2.25 ± 0.12	2.46 ± 0.19	1.84 ± 0.06	2.05 ± 0.25	1.81 ± 0.21	2.26 ± 0.31	2.31 ± 0.11	2.60 ± 0.14
Menthyl acetate	-	-	2.92 ± 0.84	2.88 ± 0.83	-	1.33 ± 0.50	-	-	5.10 ± 0.45	4.32 ± 0.65	1.33 ± 0.28	1.71 ± 0.23
β-Bourbonene	1.06 ± 0.55	-	-	-	-	-	-	-	-	-	-	-
β-Caryophyllene	1.37 ± 0.80	1.42 ± 0.21	3.08 ± 0.50	3.29 ± 0.12	1.34 ± 0.44	1.42 ± 0.04	1.09 ± 0.16	1.02 ± 0.15	-	-	-	-
β-Copaene	4.05 ± 0.24	3.87 ± 0.61	3.52 ± 0.56	4.61 ± 0.17	1.90 ± 0.63	2.41 ± 0.11	3.13 ± 0.59	3.60 ± 0.90	1.24 ± 0.27	1.11 ± 0.20	-	-

**Table 4:** The EO composition (%) of each compound of the second harvest of the years 2022 and 2023 of the three genotypes, ‘Multimentha’ (MM), ‘Fränkische Blaue’ (FB) and ‘Apfelminze’ (AM), under control (C) and shaded (S) conditions. Data are given as the mean with the standard deviation (n = 4). Compound levels below 1% are not listed (indicated by hyphen).

	2022						2023					
Compound	AM C	AM S	FB C	FB S	MM C	MM S	AM C	AM S	FB C	FB S	MM C	MM S
Eucalyptol/ limonene	15.18 ± 0.51	13.23 ± 0.15	3.55 ± 0.12	3.21 ± 0.14	2.37 ± 0.03	2.19 ± 0.14	15.02 ± 0.27	13.31 ± 0.40	3.87 ± 0.09	3.59 ± 0.13	2.67 ± 0.17	2.49 ± 0.12
*p*-Menthone	-	-	52.47 ± 1.82	55.22 ± 2.16	64.69 ± 0.81	67.35 ± 1.03	-	-	46.41 ± 1.95	49.56 ± 3.18	63.36 ± 1.96	65.36 ± 2.13
Isomenthone	-	-	7.60 ± 0.10	7.32 ± 0.15	4.32 ± 0.05	4.30 ± 0.21	-	-	7.06 ± 0.03	6.59 ± 0.08	4.16 ± 0.05	4.07 ± 0.07
Menthofuran	-	-	1.58 ± 0.16	2.12 ± 0.15	2.70 ± 0.19	2.29 ± 0.76	-	-	1.67 ± 0.21	1.25 ± 0.10	2.81 ± 0.26	2.12 ± 0.25
Menthol isomer A	-	-	1.84 ± 0.15	1.73 ± 0.20	2.50 ± 0.22	2.62 ± 0.42	-	-	2.43 ± 0.27	1.83 ± 0.27	1.95 ± 0.22	1.83 ± 0.23
Menthol isomer B	-	-	23.36 ± 2.55	21.09 ± 1.75	14.38 ± 1.16	13.16 ± 0.70	-	-	29.75 ± 2.10	26.52 ± 3.34	17.68 ± 1.79	16.09 ± 2.25
Dihydro- carvone	8.62 ± 1.88	8.24 ± 1.51	-	-	-	-	2.49 ± 0.77	2.38 ± 0.92	-	-	-	-
1,6-Dihydrocarveol	-	-	-	-	-	-	-	-	-	-	-	-
Pulegone	-	-	1.59 ± 0.15	1.03 ± 0.40	2.67 ± 0.39	2.32 ± 0.39	-	-	1.09 ± 0.34	-	1.83 ± 0.28	-
Carvone	67.29 ± 1.58	70.73 ± 1.40	-	-	-	-	73.46 ± 1.08	75.11 ± 1.32	-	-	-	-
Piperitone	-	-	1.56 ± 0.03	1.59 ± 0.04	1.91 ± 0.09	2.03 ± 0.14	-	-	1.27 ± 0.04	1.34 ± 0.03	1.59 ± 0.12	1.78 ± 0.08
Menthyl acetate	-	-	-	-	-	-	-	-	-	-	-	-
β-Bourbonene	-	-	-	-	-	-	-	-	-	-	-	-
β-Caryophyllene	1.45 ± 0.21	1.01 ± 0.11	1.49 ± 0.54	1.23 ± 0.26	-	-	1.61 ± 0.06	1.49 ± 0.13	1.38 ± 0.38	1.98 ± 0.29	-	-
β-Copaene	2.33 ± 0.33	2.19 ± 0.37	-	1.15 ± 0.22	-	-	3.17 ± 0.54	3.86 ± 1.38	1.25 ± 0.35	2.52 ± 0.49	-	1.49 ± 0.15

**Table 5:** The EO composition (%) of the third harvest of the years 2022 and 2023 of the three genotypes, ‘Multimentha’ (MM), ‘Fränkische Blaue’ (FB) and ‘Apfelminze’ (AM), under control (C) and shaded (S) conditions. Data are given as the mean with the standard deviation (n = 4). Compound levels below 1% are not listed (indicated by hyphen).

	2022						2023					
Compound	AM C	AM S	FB C	FB S	MM C	MM S	AM C	AM S	FB C	FB S	MM C	MM S
Eucalyptol/ limonene	12.88 ± 0.35	11.55 ± 0.55	3.06 ± 0.43	2.70 ± 0.15	1.72 ± 0.16	1.65 ± 0.24	13.32 ± 0.54	10.06 ± 1.79	2.76 ± 0.32	2.91 ± 0.22	1.74 ± 0.14	1.48 ± 0.19
*p*-Menthone	-	-	39.85 ± 0.94	44.71 ± 1.27	55.91 ± 2.09	60.27 ± 3.12	-	-	45.56 ± 2.93	46.84 ± 2.81	58.35 ± 1.26	60.07 ± 1.00
Isomenthone	-	-	5.40 ± 0.22	5.50 ± 0.24	3.38 ± 0.12	3.59 ± 0.12	-	-	6.05 ± 0.19	6.07 ± 0.23	3.71 ± 0.11	3.54 ± 0.12
Menthofuran	-	-	-	-	-	-	-	-	3.38 ± 0.42	3.26 ± 0.84	5.07 ± 0.61	5.88 ± 0.84
Menthol isomer A	-	-	3.21 ± 0.12	2.56 ± 0.28	4.87 ± 1.89	4.11 ± 0.94	-	-	2.93 ± 0.21	2.38 ± 0.21	3.15 ± 0.30	2.64 ± 0.33
Menthol isomer B	-	-	39.57 ± 0.61	37.44 ± 0.86	29.58 ± 1.72	26.55 ± 2.52	-	-	30.98 ± 2.35	30.24 ± 2.64	22.09 ± 0.57	20.63 ± 1.51
Dihydro- carvone	11.17 ± 1.38	15.60 ± 2.67	-	-	-	-	10.35 ± 3.47	13.50 ± 4.64	-	-	-	-
1,6-Dihydrocarveol	1.38 ± 0.17	2.39 ± 0.57	-	-	-	-	-	1.23 ± 0.62	-	-	-	-
Pulegone	-	-	-	-	-	-	-	-	-	-	1.20 ± 0.11	1.50 ± 0.20
Carvone	67.34 ± 1.73	63.41 ± 2.59	-	-	-	-	68.20 ± 3.59	67.84 ± 4.25	-	-	-	-
Piperitone	-	-	1.17 ± 0.08	1.18 ± 0.13	1.26 ± 0.04	1.29 ± 0.09	-	-	1.05 ± 0.10	1.17 ± 0.13	1.21 ± 0.06	1.14 ± 0.08
Menthyl acetate	-	-	5.25 ± 0.53	4.13 ± 0.54	1.66 ± 0.58	1.42 ± 0.36	-	-	1.80 ± 0.19	1.10 ± 0.28	-	-
β-Bourbonene	-	-	-	-	-	-	-	-	-	-	-	-
β-Caryophyllene	-	-	-	-	-	-	-	-	1.32 ± 0.11	1.26 ± 0.15	-	-
β-Copaene	1.57 ± 0.12	1.75 ± 0.28	-	-	-	-	2.26 ± 0.14	2.82 ± 0.48	1.36 ± 0.09	1.72 ± 0.30	-	-

**Table 6:** Average temperature (°C) and sum of precipitation (mm) by month for 2022 and 2023. Data are given as mean with standard deviation.

	2022		2023	
Month	Temperature [°C]	Precipitation [mm]	Temperature [°C]	Precipitation [mm]
January	3.5 ± 3.0	40.7	4.5 ± 4.7	40.8
February	5.9 ± 2.3	48.4	4.8 ± 3.6	21.0
March	6.6 ± 3.3	15.1	6.8 ± 4.2	66.0
April	8.9 ± 3.6	36.4	8.4 ± 2.6	46.0
May	15.3 ± 3.1	73.8	13.4 ± 2.3	73.2
June	18.3 ± 2.9	78.4	19.5 ± 2.5	44.0
July	19.6 ± 3.0	5.4	19.1 ± 2.5	60.3
August	21.0 ± 2.5	8.1	18.4 ± 2.8	77.6
September	14.5 ± 4.2	70.3	18.1 ± 3.1	40.4
October	13.7 ± 2.5	32.2	13.3 ± 4.0	53.6
November	8.3 ± 2.8	38.4	7.0 ± 3.4	72.7
December	3.1 ± 6.2	45.8	5.8 ± 3.8	48.6

**Table 7:** UV-A and UV-B (W/m^2^) and PAR (PPFD) measurements of control conditions and shading in the years 2022 and 2023. Data are given as means with standard deviations.

	2022		2023	
	Control	Shaded	Control	Shaded
UV-A (W/m^2^)	31.4 ± 8.3	16.5 ± 5.8	29.8 ± 12.1	15.1 ± 6.6
UV-B (W/m^2^)	0.7 ± 0.3	0.4 ± 0.2	0.7 ± 0.3	0.4 ± 0.2
PAR (PPFD)	1430 ± 434	772 ± 277	1331 ± 613	684 ± 335

**Table 8:** Categorizations of the measurements with the corresponding dates and day of the year (DOY) information.

2022			2023		
Date	Measurement	Day of the Year	Date	Measurement	Day of the Year
11 May	1	131	19 April	1	109
17 May	2	137	26 April	2	116
26 May	3	146	03 May	3	123
01 June	4	152	10 May	4	130
07 June	5	158	07 June	5	158
14 June	6	165	14 June	6	165
21 June	7	172	21 June	7	172
29 June	8	180	28 June	8	178
20 July	9	201	05 July	9	186
26 July	10	207	13 July	10	194
03 August	11	215	20 July	11	201
09 August	12	220	10 August	12	222
06 September	13	249	17 August	13	229
13 September	14	256	23 August	14	235
21 September	15	264	31 August	15	243
29 September	16	272	07 September	16	250
05 October	17	278	14 September	17	257
11 October	18	284	21 September	18	264

## Data Availability

The data presented in this study are available on request from the corresponding author.

## Supplementary Materials

The following supporting information can be downloaded at: https://www.mdpi.com/article/10.3390/plants13223155/s1, Table S1: Regression equations of the selected measurements of the years 2022 and 2023.
