# Supplementary material for: Essential Oil Composition and Physiology of Three Mentha Genotypes Under Shaded Field Conditions
Source: Plants (Basel). 2024 Nov 9;13(22):3155. doi: 10.3390/plants13223155 (PMC11597729; doi:10.3390/plants13223155)
Supplement: Supplementary file 1 [file plants-13-03155-s001.zip › plants-3293379-supplementary.pdf]

Table S1. Regression equations of the selected measurements of the years 2022 and 2023.

Year	Measurement	Genotype	Treatment	Equitation
2022	9	'Apfelminze'	Control	$-0.8617 + 0.2692 * X - 8.734e^{-5} * X^2 + 1.005e^{-8} * X^3$
2022	9	'Apfelminze'	Shaded	$-1.256 + 0.2579 * X - 9.616e^{-5} * X^2 + 1.179e^{-8} * X^3$
2022	9	'Fr. Blaue'	Control	$-3.418 + 0.2693 * X - 9.109e^{-5} * X^2 + 1.094e^{-8} * X^3$
2022	9	'Fr. Blaue'	Shaded	$-5.215 + 0.2773 * X - 9.305e^{-5} * X^2 + 1.061e^{-8} * X^3$
2022	9	'Multimentha'	Control	$1.403 + 0.2284 * X - 8.33e^{-5} * X^2 + 1.014e^{-8} * X^3$
2022	9	'Multimentha'	Shaded	$-3.537 + 0.2706 * X - 9.801e^{-5} * X^2 + 1.205e^{-8} * X^3$
2022	12	'Apfelminze'	Control	$0.122 + 0.2467 * X - 8.894e^{-5} * X^2 + 1.12e^{-8} * X^3$
2022	12	'Apfelminze'	Shaded	$-1.703 + 0.2629 * X - 0.000108 * X^2 + 1.492e^{-8} * X^3$
2022	12	'Fr. Blaue'	Control	$-0.474 + 0.2462 * X - 9.8e^{-5} * X^2 + 1.309e^{-8} * X^3$
2022	12	'Fr. Blaue'	Shaded	$-0.6569 + 0.2427 * X - 9.727e^{-5} * X^2 + 1.282e^{-8} * X^3$
2022	12	'Multimentha'	Control	$1.542 + 0.2343 * X - 9.912e^{-5} * X^2 + 1.386e^{-8} * X^3$
2022	12	'Multimentha'	Shaded	$2.519 + 0.2309 * X - 9.965e^{-5} * X^2 + 1.379e^{-8} * X^3$
2022	16	'Apfelminze'	Control	$2.708 + 0.1769 * X - 8.676e^{-5} * X^2 + 1.356e^{-8} * X^3$
2022	16	'Apfelminze'	Shaded	$0.836 + 0.141 * X - 7.504e^{-5} * X^2 + 1.204e^{-8} * X^3$
2022	16	'Fr. Blaue'	Control	$-1.022 + 0.1854 * X - 9.106e^{-5} * X^2 + 1.397e^{-8} * X^3$
2022	16	'Fr. Blaue'	Shaded	$0.495 + 0.166 * X - 8.436e^{-5} * X^2 + 1.333e^{-8} * X^3$
2022	16	'Multimentha'	Control	$1.08 + 0.1784 * X - 8.658e^{-5} * X^2 + 1.297e^{-8} * X^3$
2022	16	'Multimentha'	Shaded	$1.115 + 0.1637 * X - 8.815e^{-5} * X^2 + 1.413e^{-8} * X^3$
2023	7	'Apfelminze'	Control	$0.04937 + 0.2509 * X - 8.939e^{-5} * X^2 + 1.055e^{-8} * X^3$
2023	7	'Apfelminze'	Shaded	$-1.423 + 0.2749 * X - 0.000113 * X^2 + 1.551e^{-8} * X^3$

2023	7	'Fr. Blaue'	Control	$4.387 + 0.2078 * X - 9.438e^{-5} * X^2 + 1.402e^{-8} * X^3$
2023	7	'Fr. Blaue'	Shaded	$-1.331 + 0.2733 * X - 0.000111 * X^2 + 1.486e^{-8} * X^3$
2023	7	'Multimentha'	Control	$2.3 + 0.2288 * X - 9.672e^{-5} * X^2 + 1.329e^{-8} * X^3$
2023	7	'Multimentha'	Shaded	$-0.1664 + 0.2658 * X - 0.000118 * X^2 + 1.673e^{-8} * X^3$
2023	14	'Apfelminze'	Control	$-2.45 + 0.2545 * X - 0.000106 * X^2 + 1.421e^{-8} * X^3$
2023	14	'Apfelminze'	Shaded	$-0.4985 + 0.2511 * X - 0.000115 * X^2 + 1.698e^{-8} * X^3$
2023	14	'Fr. Blaue'	Control	$-1.589 + 0.2461 * X - 0.000102 * X^2 + 1.428e^{-8} * X^3$
2023	14	'Fr. Blaue'	Shaded	$-0.7092 + 0.2408 * X - 0.000102 * X^2 + 1.406e^{-8} * X^3$
2023	14	'Multimentha'	Control	$1.837 + 0.2211 * X - 0.000104 * X^2 + 1.553e^{-8} * X^3$
2023	14	'Multimentha'	Shaded	$3.116 + 0.2158 * X - 0.000107 * X^2 + 1.628e^{-8} * X^3$
2023	17	'Apfelminze'	Control	$0.3764 + 0.2337 * X - 0.000107 * X^2 + 1.602e^{-8} * X^3$
2023	17	'Apfelminze'	Shaded	$4.099 + 0.2106 * X - 0.000106 * X^2 + 1.634e^{-8} * X^3$
2023	17	'Fr. Blaue'	Control	$-1.438 + 0.2378 * X - 0.000101 * X^2 + 1.372e^{-8} * X^3$
2023	17	'Fr. Blaue'	Shaded	$1.452 + 0.2171 * X - 0.000101 * X^2 + 1.499e^{-8} * X^3$
2023	17	'Multimentha'	Control	$3.219 + 0.2235 * X - 0.000104 * X^2 + 1.498e^{-8} * X^3$
2023	17	'Multimentha'	Shaded	$8.245 + 0.1607 * X - 8.818e^{-5} * X^2 + 1.445e^{-8} * X^3$
